# Kinetics of blood neutrophil and lymphocyte count in critically ill neutropenic patients with hematological malignancies

**DOI:** 10.1186/s13054-021-03740-0

**Published:** 2021-08-30

**Authors:** Andry Van de Louw

**Affiliations:** Division of Pulmonary and Critical Care Medicine, Penn State Health Milton S Hershey Medical Center, 500 University Dr, Hershey, PA USA

In critically ill patients with hematological malignancies (HM), neutropenia and lymphopenia often coexist, whether related to HM or critical illness immunoparalysis [1]. Both cytopenias expose patients to increased risk of infection and possibly mortality [1, 2]. However, little is known about the kinetics of neutrophil and lymphocyte count in neutropenic patients, which might affect the risk of serious infections in this vulnerable population.

We aimed to describe the course of blood neutrophil and lymphocyte count over time in adult patients with HM admitted to the ICU with severe neutropenia (neutrophil count ≤ 0.5 × 10^9^/L within 24 h of ICU admission) between 2008 and 2018.

All neutrophil and lymphocyte counts during hospital admission were collected along with demographics, underlying HM, main laboratory values on ICU admission, vital organ support in ICU and hospital mortality.

We included 210 patients (59% males, median age 61 [IQR 47–67] years), mostly with acute leukemias (66%) and lymphomas (22%) (Table 1). Vasopressors, invasive mechanical ventilation and renal replacement therapy were required in 44%, 42% and 8% of patients, respectively. Hospital mortality was 40%.

**Table 1:** Patients’ main characteristics

	Patients (*n* = 210)
Male gender, *n* (%)	124 (59%)
Age (years)	61 (47–67)
Hematological malignancy, *n* (%)	
Acute leukemia	139 (66%)
Non-Hodgkin lymphoma	41 (20%)
Hodgkin lymphoma	5 (2%)
Multiple myeloma	14 (7%)
Other	11 (5%)
Laboratory on ICU admission	
Neutrophil count, × 10^9^/L	0.06 (0.02–0.28)
Lymphocyte count, × 10^9^/L	0.14 (0.04–0.42)
White blood cell count, × 10^9^/L	0.34 (0.07–1.16)
Platelet count, × 10^9^/L	24 (13–43)
Hemoglobin, g/dL	7.9 (7.1–8.8)
Creatinine, mg/dL	1.13 (0.75–1.61)
HCO_3_^−^, mmol/L	22 (19–26)
Lactate, mmol/L (*n* = 117)	1.8 (1.2–3.4)
Vital organ support in the ICU	
Vasopressors, *n* (%)	92 (44%)
Mechanical ventilation, *n* (%)	88 (42%)
Renal replacement therapy, *n* (%)	17 (8%)
Hospital mortality, *n* (%)	83 (40%)

Results are presented as median (interquartile range) and number (percentage) for continuous and categorical variables, respectively

Figure 1a displays the course of blood neutrophil and lymphocyte counts over time: The mean neutrophil count sharply decreased to reach its nadir (mean ± SEM 0.19 ± 0.17 × 10^9^/L) on ICU admission with subsequent re-increase. The duration of severe neutropenia was 6 (3–12) days for survivors. By contrast, the lymphocyte count followed a flat curve with mean values consistently ≤ 0.5 × 10^9^/L throughout admission. The mean ± SEM lymphocyte count on ICU admission was 0.35 ± 0.02 × 10^9^/L. Severe lymphopenia (≤ 0.5 × 10^9^/L) was present on ICU admission in 137 patients, and its duration was 10 (4–17) days for survivors. Severe lymphopenia persisted for more than 3 and 7 days in 157 (75%) and 109 (52%) patients, respectively.

**Fig. 1:**
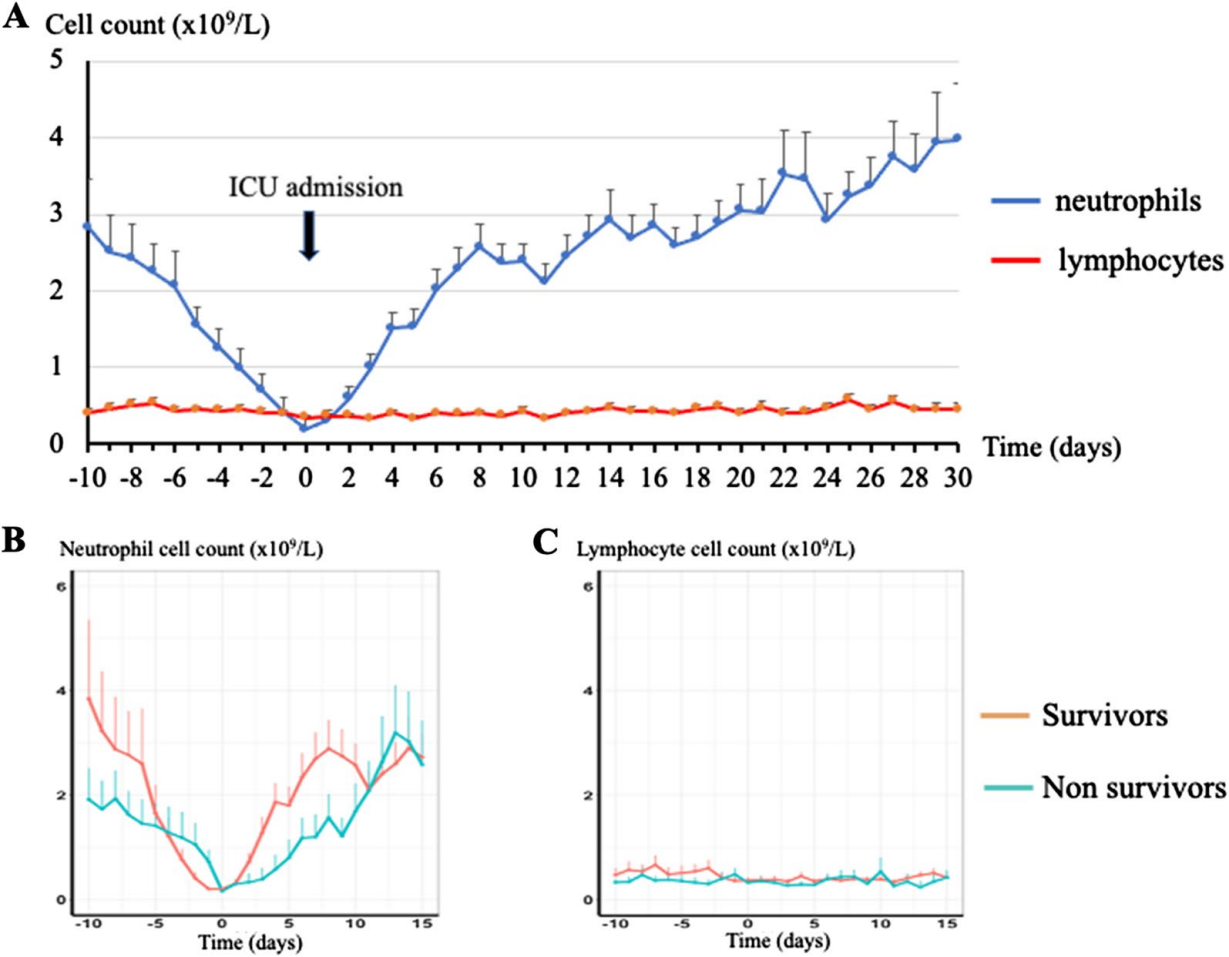
Kinetics of blood neutrophil and lymphocyte counts in 210 critically ill neutropenic patients. **a** Mean + SEM of blood neutrophil (blue) and lymphocyte (red) counts over time, from 10 days prior to ICU admission to 30 days after ICU admission. ICU admission is at day 0. **b** Mean + SEM of blood neutrophil count over time, from 10 days prior to ICU admission to 15 days after ICU admission, for survivors (red) and non-survivors (blue). ICU admission is at day 0. **c** Mean + SEM of blood lymphocyte count over time, from 10 days prior to ICU admission to 15 days after ICU admission, for survivors (red) and non-survivors (blue). ICU admission is at day 0

The slope of neutrophil count decline and recovery was sharper in survivors than in non-survivors (Fig. 1b), whereas no discernable difference was observed for lymphocyte count (Fig. 1c). In survivors, neutrophil count was significantly higher between day 2 and day 5, whereas no difference with non-survivors was observed at any time for lymphocyte count.

The lymphopenia in our patients was more severe than in general ICU populations, where lymphocyte counts of 0.5–1 × 10^9^/L were reported [3, 4]. Whether this is related to HM and chemotherapy or reflects a more pronounced critical illness immunoparalysis remains unclear. In a similar analysis in 407 ICU patients with HM but without neutropenia, we observed a similar flat curve for the lymphocyte count throughout admission but at a level close to 0.8 × 10^9^/L (not shown), similar to reports in unselected ICU patients [3, 4]. Second, while lymphopenia has been well documented in critically ill patients [3, 4], most studies focused on ICU admission values with little information on kinetics. Lymphopenia in our population was very prolonged. We did not observe a difference in absolute values or kinetics between survivors and non-survivors, but studies are warranted to ascertain the effect of lymphopenia in this specific population. Finally, the median neutropenia duration was 6 days in our patients with a faster recovery in survivors. Another study reported a similar duration of neutropenia and lower mortality in patients who experienced neutropenia recovery in the ICU [5].

Several important points were not addressed in this study and are avenues for future research: (1) the association between lymphocyte kinetics and infections, (2) the qualitative aspect of neutrophil and lymphocyte function, which may remain altered beyond count recovery [6], and (3) the effect of hematopoietic growth factor administration in this setting.

In summary, critically ill neutropenic patients experience severe and prolonged lymphopenia. Our data suggest that survival might be associated with faster neutropenia recovery.

## Data Availability

All data generated or analyzed during this study are included in this published article.

## Abbreviations

HM: Hematological malignancies
ICU: Intensive care unit
SEM: Standard error of the mean
